# Novice providers’ success in performing lumbar puncture: a randomized controlled phantom study between a conventional spinal needle and a novel bioimpedance needle

**DOI:** 10.1186/s12909-024-05505-z

**Published:** 2024-05-10

**Authors:** Helmiina Lilja, Maria Talvisara, Vesa Eskola, Paula Heikkilä, Harri Sievänen, Sauli Palmu

**Affiliations:** 1 Faculty of Medicine and Health Technology, Tampere University, Arvo Ylpön katu 34, Tampere, 33520 Finland; 2 Tampere Center for Child, Adolescent and Maternal Health Research, Faculty of Medicine and Health Technology, Tampere University, Arvo Ylpön katu 34, Tampere, 33520 Finland; 3 Tampere University Hospital, Elämänaukio 2, Tampere, 33520 Finland; 4 Injeq Plc, Biokatu 8, Tampere, Tampere, 33520 Finland

**Keywords:** Lumbar puncture, Spinal needle, Clinical skill, Bioimpedance, Training

## Abstract

**Background:**

Lumbar puncture (LP) is an important yet difficult skill in medical practice. In recent years, the number of LPs in clinical practice has steadily decreased, which reduces residents’ clinical exposure and may compromise their skills and attitude towards LP. Our study aims to assess whether the novel bioimpedance needle is of assistance to a novice provider and thus compensates for this emerging knowledge gap.

**Methods:**

This randomized controlled study, employing a partly blinded design, involved 60 s- and third-year medical students with no prior LP experience. The students were randomly assigned to two groups consisting of 30 students each. They performed LP on an anatomical lumbar model either with the conventional spinal needle or the bioimpedance needle. Success in LP was analysed using the independent samples proportion procedure. Additionally, the usability of the needles was evaluated with pertinent questions.

**Results:**

With the conventional spinal needle, 40% succeeded in performing the LP procedure, whereas with the bioimpedance needle, 90% were successful (*p* < 0.001). The procedures were successful at the first attempt in 5 (16.7%) and 15 (50%) cases (*p* = 0.006), respectively. Providers found the bioimpedance needle more useful and felt more confident using it.

**Conclusions:**

The bioimpedance needle was beneficial in training medical students since it significantly facilitated the novice provider in performing LP on a lumbar phantom. Further research is needed to show whether the observed findings translate into clinical skills and benefits in hospital settings.

## Background

Lumbar puncture (LP) is one of the essential skills of physicians in medical practice, especially in the fields of neurology, neurosurgery, emergency medicine and pediatrics. It is one of the procedures that medical students practice in their training. LP is an important clinical procedure for diagnosing neurological infections and inflammatory diseases and excluding subarachnoid hemorrhage [1]. LP can also be used for examining the spread of cancer cells to the central nervous system in diagnosing acute lymphoblastic leukemia (ALL) and for delivering intrathecal administration of chemotherapy in patients with ALL [2]. In recent years, the number of LPs in clinical practice has steadily decreased [3, 4]. Over the past decade, a 37% decrease in LPs was observed across US children’s hospitals [3]. Similar trends have also been observed in emergency medicine [4]. Stricter criteria in practice guidelines, changes in patient demographics, and development in medical imaging have likely contributed to this decrease. This trend presumably reduces residents’ clinical exposure and may compromise their skills and attitude towards LP.

When performed by an experienced physician, LP is a relatively safe procedure, albeit not always straightforward or free from complications [4]. The spinal needle used in LP is thin and flexible, making its insertion into the spinal canal without seeing the location of the needle tip or destination challenging. The physician performing the procedure must master the specific lumbar anatomy to avoid complications [5]. The LP technique is not the only thing that matters, but patients’ size and comfort also affect the success of the procedure [6]. Hence, a practitioner lacking adequate experience in LP should be appropriately supervised when performing the procedure [4]. Nevertheless, there are situations in which such supervision is not possible.

Little experience in performing LPs may require more attempts to obtain cerebrospinal fluid (CSF) samples [7]. Because of several attempts, blood can be introduced to CSF and result in a traumatic LP. Success at the first attempt is associated with a lower incidence of traumatic LPs [2, 8–12]. A bloody CSF sample complicates the diagnostics [8]. It has also been shown that a high number of attempts increases the incidence of postdural puncture headache (PDPH), the most common complication of LP, in addition to other adverse effects [9].

Considering the possible complications and difficulties of performing LP, a concern arises regarding whether inexperienced physicians can perform LP with adequate confidence and safety. The use of a novel bioimpedance-based spinal needle system could offer a solution. This needle provides real-time feedback from the needle tip when penetrating the lumbar tissues and informs the physician when the needle tip reaches CSF with an audio-visual alarm. This information may make performing the LP procedure smoother, thus decreasing the incidence of the most common complications [13]. A bioimpedance-based spinal needle system has been recently found clinically feasible in LPs among adults, adolescents, and children, including neonates [2, 14, 15].

The current phantom study aimed to assess whether the novel needle technology can compensate for the lack of experience when a medical student performs LP for the first time. In particular, we compared the performance of the bioimpedance spinal needle and conventional spinal needle in terms of the overall success rate of the LP procedure, success rate at the first attempt, duration of the procedure, and number of stylet removals. We hypothesized that novice users would find the bioimpedance needle more useful in performing LPs than a conventional spinal needle. If so proven, the use of this novel device can contribute to training medical students in this important skill and facilitate situations when an inexperienced physician needs to perform LP without the supervision and guidance of an experienced physician [4].

## Methods

We planned to recruit 60 medical students from Tampere University in this randomized controlled trial. Students who were studying medicine for their third year or less were considered eligible for the study. At this stage of studies, they were expected to have no clinical experience and be thus naïve in performing an LP. All students had the same baseline knowledge regarding lumbar spine anatomy.

The participants were recruited by sending an invitation e-mail to all potentially eligible medical students. The email provided information about the study. Of the 177 students who responded to the invitation, 60 students were included on a first-come-first-serve basis. The participants were rewarded with a 10€ voucher to the university campus cafeteria.

Randomization lists in blocks of six were generated for two groups (A and B) before recruitment by an independent person who was not involved in recruitment or data collection. Participants assigned to group A used a conventional spinal needle (90 mm long 22G Quincke-type needle), and those to group B used the bioimpedance needle system (IQ-Tip system with a 90 mm long IQ-Tip needle, Injeq Plc, Tampere, Finland).

The study LPs were performed on an adult-size anatomical lumbar phantom (Blue Phantom BPLP2201, CAE Healthcare, FL, USA) intended for medical training and practising. The phantom is made of a tissue-simulating elastomer material that looks and feels like human soft tissue. Skeletal structures made of hard material and a plastic tube mimicking the spinal canal are embedded in the phantom. The saline inside the tube mimics CSF and is under hydrostatic pressure. The phantom offers a relatively realistic feel in palpating the lumbar anatomy and getting haptic feedback from the advancing needle.

The study LPs were performed in February 2023 in ten different sessions, with 6 participants in each session. Two separate rooms were used to conduct the study. The participants were first admitted to a waiting room and then separately to another room where each student performed the study LP with the assigned spinal needle under supervision (HL and MT). By having these two rooms, we ensured that no information was exchanged after or during the procedure.

Before the study LPs, the participants were shown an instructional video on how to perform an LP from the widely used Finnish medical database Terveysportti [16] and a video on the operation of the bioimpedance needle [13]. The first video (duration 3 min) describes the indications, contraindications and a step-by-step instruction on how the procedure is performed. The latter is a 25- second animation showing how the bioimpedance system operates and guides the procedure. In addition, the supervisor gave each participant the following instructions before starting the study LP: *When you think you have reached the subarachnoid space, remove the stylet from the needle. If you are in the correct place, the fluid will start flowing from the needle. You may redirect the needle as many times as you wish, but you are only allowed to remove the needle and do a new attempt five times. Please wait a while when you have removed the stylet because it may take a while before the fluid starts dropping.* These instructions were given to all participants irrespective of the study group to standardize the information in all sessions.

After watching the videos and listening to the instructions, the participants became aware of their assigned study group. Participants were allowed five attempts, while redirections of the needle and stylet removals could be performed as many times as needed. We measured the duration of the LP procedure and collected data on the number of stylet removals, the number of attempts, and whether the LP was successful.

The duration of the procedure was defined from the point when the needle penetrated the phantom surface to either when the first drop of fluid fell from the needle, or the participant wanted to stop or had used all five attempts. There was no maximum time for completing the LP procedure. The procedure was defined as successful if the participant succeeded in obtaining a drop of fluid from the needle.

In addition, seven relevant statements to this study were chosen from the System Usability Scale (SUS) [17], which is an industry standard for evaluating the usability of various devices and systems. The seven statements, slightly modified from the original statements, are shown in Table 1. After performing the study LP and irrespective of their success, all participants were asked to respond to the statements using a 5-point Likert scale (1 = strongly disagree, 5 = strongly agree).

**Table 1:** The statements used in the usability questionnaire. Modified from the original System Usability Scale [17] for this study

Statement	Strongly disagree				Strongly agree
1. I could use the tested spinal needle regularly. (Q1)	1	2	3	4	5
2. Lumbar puncture was easy to perform with the tested spinal needle. (Q3)	1	2	3	4	5
3. Learning to use the tested spinal needle needs support from an experienced user. (Q4)	1	2	3	4	5
4. Most people would learn to use the tested spinal needle very quickly. (Q7)	1	2	3	4	5
5. The tested needle was cumbersome to use. (Q8)	1	2	3	4	5
6. I felt myself confident using the tested spinal needle. (Q9)	1	2	3	4	5
7. I needed to learn a lot of things before I could use the tested spinal needle. (Q10)	1	2	3	4	5

Qx refers to the original SUS question x.

### Statistical analysis

For the estimation of statistical power, we assumed that the overall success rate would be 60% with the conventional needle (group A) and 90% with the bioimpedance needle (group B). Then, the sample size of 60 participants divided randomly into two equal-sized groups would be sufficient to detect a between-group at a significance level of *p* < 0.05 and with 80% statistical power if such a difference truly exists.

Overall success in performing the lumbar puncture and success at the first attempt in the groups were analysed by the independent samples proportion procedure. The median number of attempts and stylet removals in the successful procedures were compared by independent samples Mann‒Whitney U test. Responses to the seven usability statements were compared by this test as well.

Statistical analyses were performed with IBM SPSS Statistics for Windows, version 29.0 (IBM Corp., Armonk, NY, USA). A p value less than 0.05 was considered statistically significant.

## Results

Sixty medical students were randomly assigned into two groups, 30 performing the LP procedure on the lumbar phantom using a conventional spinal needle and 30 using the bioimpedance needle. None of the participants had previous experience in performing an LP.

With the conventional spinal needle (group A), 12 out of 30 participants (40%) succeeded in performing the LP procedure, whereas with the bioimpedance needle (group B), 27 out of 30 participants (90%) were successful (*p* < 0.001). The procedures were successful at the first attempt in 5 (16.7%) and 15 (50%) cases (*p* = 0.006), respectively.

**Fig. 1:**
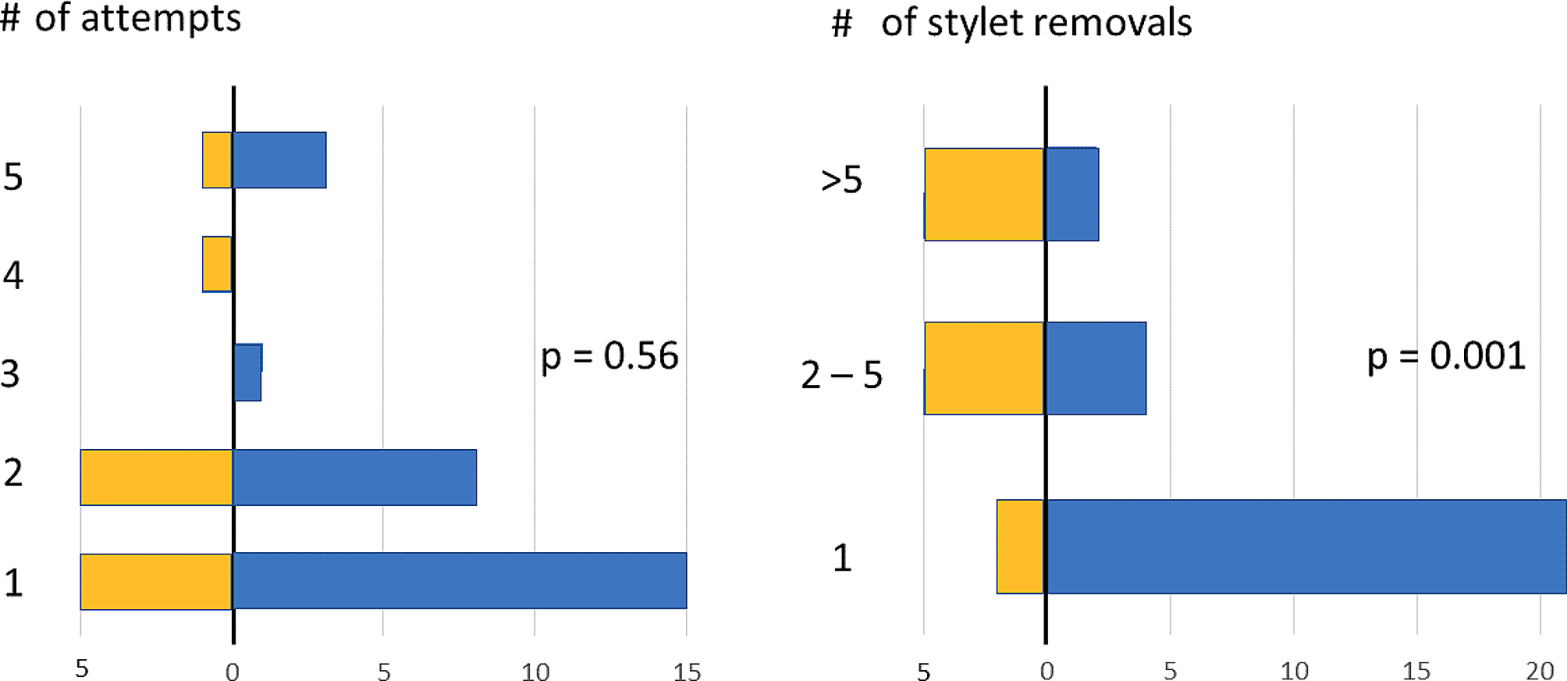
Distributions of the number of attempts in successful LP procedures (left panel) with the conventional spinal needle (group A, yellow bars) and with the bioimpedance needle (group B, blue bars). Respective distributions of the number of stylet removals (right panel) in groups A and B

Figure 1 illustrates the number of attempts and stylet removals in the study groups. Regarding the success of the procedure at any attempt, the median number of attempts was 2 (range 1–5) for the conventional needle and 1 [1–5] for the bioimpedance needle (*p* = 0.56).

In the successful procedures, the median number of stylet removals was 4 [1–26] and 1 (1–33) (*p* = 0.001), respectively. The mean duration of a successful procedure was 3:51 (SD 3:43) with the conventional needle and 1:59 (2:25) with the bioimpedance needle (*p* = 0.068).

**Fig. 2:**
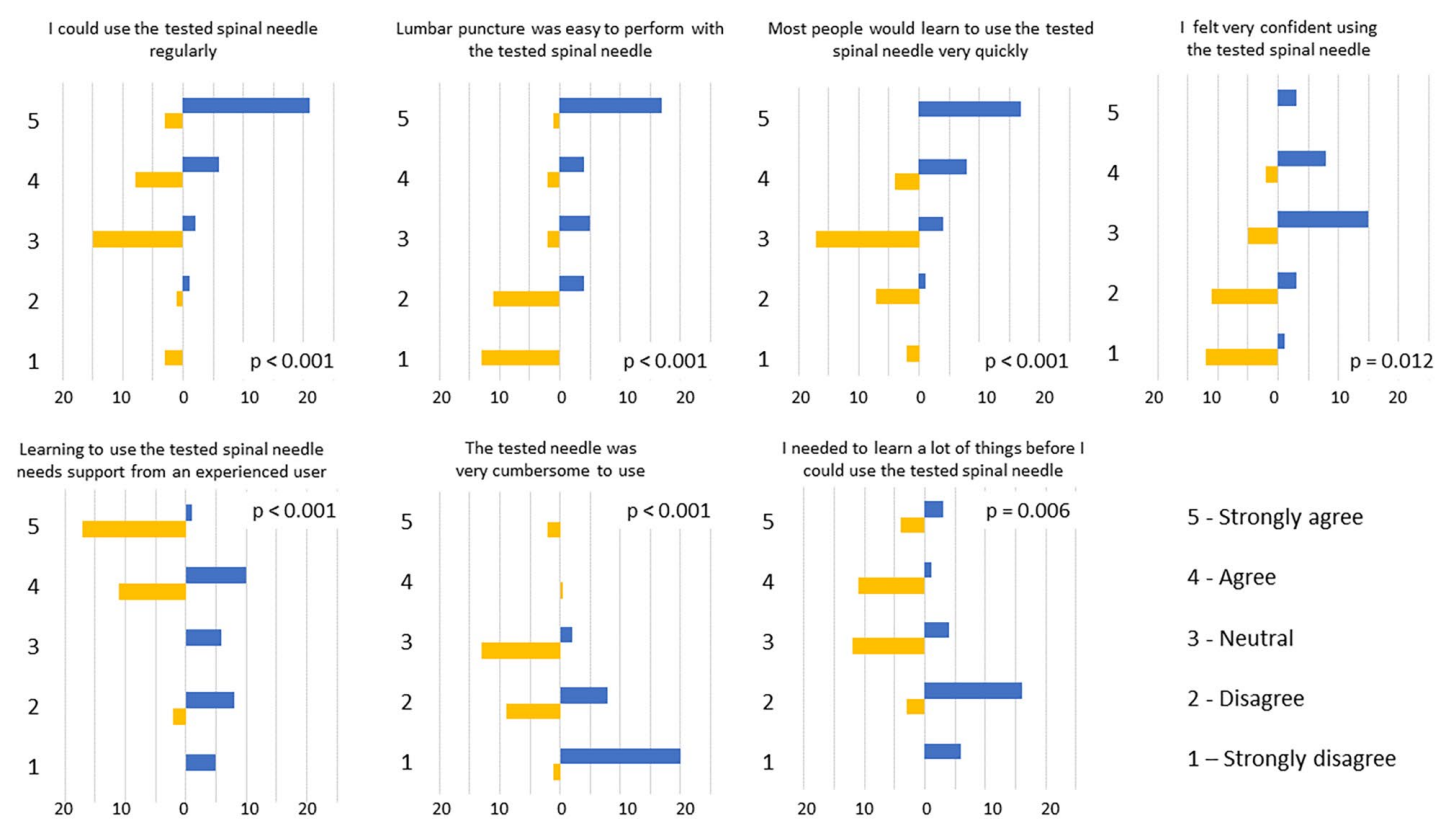
After performing the LP, the provider answered seven statements about the usability of the needle in question on a scale of 1 (strongly disagree) to 5 (strongly agree). Distributions of responses to every seven usability statements in group A (conventional spinal needle, yellow bars) and in group B (bioimpedance needle, blue bars) using the System Usability Scale (SUS)

The responses to the seven usability statements are illustrated in Fig. 2. Regarding the statements on regular use, ease of use, need for support from an experienced user, learning to use, and cumbersomeness, the responses differed significantly between groups, consistently favouring the bioimpedance needle (*p* < 0.001). Regarding the feeling of confidence in use, the responses significantly favoured the bioimpedance needle (*p* = 0.012). Likewise, the responses significantly favoured the bioimpedance needle to less need to learn many things before its use.

## Discussion

The decline in the number of LPs during the last decade [3, 4] likely weakening the practical knowledge and skills of novice physicians served as the rationale for the current study. Using a solid randomized controlled study design, we assessed whether bioimpedance-based tissue detection technology could help an inexperienced provider perform LP. Our study was conducted among early-stage medical students who had no previous experience with LPs. Following our hypothesis, we found that the use of a bioimpedance needle in simulated phantom LPs was useful to novice providers. The bioimpedance needle decreased not only the number of attempts to achieve a successful LP but also its time, in addition to the significantly lower number of stylet removals during the procedure. Furthermore, the usability of the bioimpedance needle was found to be significantly better than that of the spinal needle used currently in clinical practice.

The users of the bioimpedance needle found the novel device easy and intuitive to learn and use while feeling more confident in performing LP compared to those using the conventional needle. They also expressed their interest in using the bioimpedance needle regularly. It is recalled that the present providers were all novices without earlier experience in LP, and therefore, the observed between-group differences in performance could have been smaller with more experienced providers.

Of common bedside procedures in clinical practice, LP was recently found to be associated with the lowest baseline levels of experience and confidence among 4^th−^ to 6th -year medical students. However, a single seminar with standardized simulation training brought more confidence to the LP procedure among these students [18]. Other recent studies have also shown that simulation-based education can improve procedural competence and skills in performing LP [19–22]. In these studies, the participants had more experience than in our study, but the benefits of simulation-based learning were significant. A recent study assessing a mixed reality simulator found this approach helpful in learning of LP among residents, faculty, interns, and medical students, approximately 60% having no previous experience in LP [23]. After mixed reality training, the success rate of LP increased while the time of the procedure decreased [23], which is in line with our findings. Virtual reality-based training in LP learning has also been studied, and it might have beneficial results in the provider’s skills and confidence [24, 25]. All these findings speak for the utility of various simulation approaches in adopting essential (new) clinical skills for LP at different stages of medical studies and careers.

Lumbar puncture is commonly considered a difficult and possibly frightening procedure to perform. In addition to the physician’s experience and skills, there are other factors that affect the success of LP, including patient size, spinal deformities, lumbar anatomy, cooperation and comfort [6]. Occasionally, a physician may have to insert the needle more than once to succeed in LP. However, repeated attempts are associated with several complications, such as PDPH and traumatic LP [7, 10–12, 26–28]. In our study, the median number of attempts was two for the conventional spinal needle and one for the bioimpedance needle. The low number of attempts may have also contributed to the low incidence of traumatic LP and PDPH observed in pediatric patients with leukemia, whose intrathecal therapy was administered using the bioimpedance needle [15]. Since the basic use of a bioimpedance needle is virtually similar to that of a conventional spinal needle with no need for additional devices (e.g., ultrasound imaging), it may offer a notable option for effective teaching of LP among medical students. Its real-time CSF detection ability is likely to consolidate the learning experience and increase confidence in one’s skills.

In this study, we found a significantly higher success rate and confidence in procedural skills of medical students associated with using the bioimpedance needle compared to the conventional spinal needle. Should these benefits translate into the real clinical world and manifest as a lower incidence of failed LP procedures and procedure-related complications, a higher incidence of high-quality CSF samples, a lower need for repeated procedures, a lower need for experienced and more expensive physicians to supervise, perform, or complete the LP procedure, substantial savings in the total costs of the lumbar puncture procedure are possible despite the initially higher unit cost of the bioimpedance needle system compared to conventional spinal needles. Further clinical studies on the benefits of the bioimpedance needle system in clinical LP procedures are needed to confirm these speculations.

The major strengths of the present study are the randomized controlled, partly blinded design and adequate sample size. The random assignment of participants to study groups and data analysis were performed by an independent person who was not involved in recruitment or data collection. The participants received the same instructions and information before performing their assigned LP procedure and were asked not to study LP in advance to keep the participants as naïve in performing LP as possible. Obviously, we could not control for this and have full certainty about the prior information on retrieval of the participants. However, the participants were not told before the study session which type of spinal needle they would use in their assigned LP.

During the LP sessions, there were a few technical issues concerning the lumbar phantom and bioimpedance needle. First, since the pressure inside the phantom spinal canal (plastic tube) affects the fluid flow through the needle, we attempted to keep the height of the hydrostatic saline column constant by adding new saline as needed, but slight variation in pressure may have occurred, and concerned all study LP procedures. Second, when the plastic tube and surrounding phantom material are pierced multiple times in succession, it is possible that the leakage of saline moistens the rubbery material and increases markedly its electrical conductivity despite the self-healing property of the material. Had this happened, consequent false detections may have led to unnecessary removals of the stylet in the LP procedures performed with the bioimpedance needle system. Therefore, as a precaution, the maximum number of participants at each session was limited to six to mitigate the risk of moistening of material. Third, in two cases, the bioimpedance needle system did not detect saline, although the needle tip was in the correct place, confirmed by saline flow after stylet removal. This rate of missed detections in line with clinical experience [2, 15] and may be due to elastomer remnants stuck at the needle tip compromising the bioimpedance measurement and saline detection. However, despite the failed functionality, the mechanical performance of the bioimpedance needle as a spinel needle is maintained and LP could be performed as usual. Regarding the credibility of the present findings, the bioimpedance needle did not get any undue benefit from these technical issues compared to the conventional spinal needle.

Given that the participants were clinically inexperienced early-stage medical students, the study was conducted using an anatomical lumbar phantom, not on actual patients. Obviously, the haptic feedback from the phantom and anatomic variation in the lumbar region do not fully correspond to a real patient. On the other hand, the use of phantom takes off the pressure from a novice provider and possibly eases the procedure, not having to take thought on a patient’s comfort, anatomy, and condition. Although the LP procedure was performed for the first time without the guidance of an experienced physician, the users of the bioimpedance needle felt more confident and performed significantly better than those with the conventional spinal needle. If used for teaching purposes, the bioimpedance needle and the anatomical lumbar phantom could offer a positive experience of the LP procedure and raise confidence in one’s own skills before the first real patient encounter. Whether the present promising results of a phantom study would translate into improved performance in actual clinical work calls for further investigation.

## Conclusions

Lumbar puncture is a widely used but demanding procedure needed for the diagnosis and treatment of several diseases. It is relatively safe when performed correctly, but due to the decreasing trend of performed LP procedures, a concern has arisen concerning novice physicians’ expertise in LP. The bioimpedance needle could offer a solution to this problem and facilitate practical training of LP among early-stage medical students. The present randomized controlled phantom study showed that providers with no previous experience in LP perceived the bioimpedance needle as more useful, became confident, and achieved significantly higher success rates both overall and at the first attempt with fewer stylet removals compared to those using a conventional spinal needle. Further research is needed to show whether the observed findings translate into clinical skills and benefits in hospital settings.

## Data Availability

The datasets used and/or analyzed during the current study are available from the corresponding author on reasonable request.

## Abbreviations

ALL: Acute lymphoblastic leukemia
CSF: Cerebrospinal fluid
LP: Lumbar puncture
PDPH: Postdural puncture headache
